# The association between penicillin allergy and surgical site infection after orthopedic surgeries: a retrospective cohort study

**DOI:** 10.3389/fcimb.2023.1182778

**Published:** 2023-04-21

**Authors:** Tong Niu, Yuelun Zhang, Ziquan Li, Yanyan Bian, Jianguo Zhang, Yipeng Wang

**Affiliations:** ^1^Department of Orthopedics, Peking Union Medical College Hospital, Peking Union Medical College and Chinese Academy of Medical Sciences, Beijing, China; ^2^Medical Research Centre, Peking Union Medical College Hospital, Peking Union Medical College and Chinese Academy of Medical Sciences, Beijing, China

**Keywords:** alternative antibiotics, antimicrobial prophylaxis, orthopedics, penicillin allergy, surgical site infection

## Abstract

**Background:**

Cephalosporins are used as first-line antimicrobial prophylaxis for orthopedics surgeries. However, alternative antibiotics are usually used in the presence of penicillin allergy (PA), which might increase the risk of surgical site infection (SSI). This study aimed to analyze the relationship between SSI after orthopedic surgeries and PA among surgical candidates and related alternative antibiotic use.

**Methods:**

In this single-center retrospective cohort study, we compared inpatients with and without PA from January 2015 to December 2021. The primary outcome was SSI, and the secondary outcomes were SSI sites and perioperative antibiotic use. Moreover, pathogen characteristics of all SSIs were also compared between the two cohorts.

**Results:**

Among the 20,022 inpatient records, 1704 (8.51%) were identified with PA, and a total of 111 (0.55%) SSI incidents were reported. Compared to patients without PA, patients with PA had higher postoperative SSI risk (1.06%, 18/1704 vs. 0.51%, 93/18318), shown both in multivariable regression analysis (odds ratio [OR] 2.11; 95% confidence interval [CI], 1.26-3.50; p= 0.004) and propensity score matching (OR 1.84; 95% CI, 1.05-3.23; p= 0.034). PA was related to elevated deep SSI risk (OR 2.79; 95% CI, 1.47-5.30; p= 0.002) and had no significant impact on superficial SSI (OR 1.39; 95% CI, 0.59-3.29; p= 0.449). The PA group used significantly more alternative antibiotics. Complete mediation effect of alternative antibiotics on SSI among these patients was found in mediation analysis. Pathogen analysis revealed gram-positive cocci as the most common pathogen for SSI in our study cohort, while patients with PA had higher infection rate from gram-positive rods and gram-negative rods than non-PA group.

**Conclusion:**

Compared to patients without PA, patients with PA developed more SSI after orthopedic surgeries, especially deep SSI. The elevated infection rate could be secondary to the use of alternative prophylactic antibiotics

## 1 Introduction

Surgical site infection (SSI) is among the most lethal surgical complications both for surgeons and patients, leading to elevated postoperative morbidity and mortality rate (Beam and Osmon, 2018; Dagneaux et al., 2021; Sarfani et al., 2022). SSI is multifactorial, relating to patients’ age, diabetic state, nutrition status, smoking history, etc. (Ban et al., 2017), and usually requires long-term antibiotics or even secondary operation. The SSI after orthopedic surgeries could be more complicated in nature as patients are usually older in age, with more comorbidities, and receive more surgeries with internal fixation/prosthesis implantation. A study published in 2014 showed an average of $11,876 increased cost within 30 postoperative days if the patient developed SSI. This additional cost would further rise to $15,243 for orthopedic patients, only secondary to that of the neurosurgical patients (Schweizer et al., 2014), making SSI a study focus in this field.

Reducing SSI requires multi-dimensional modalities. Surgical Care Improvement Project recommended appropriate antimicrobial prophylaxis (AMP), serum glucose control, proper skin preparation, early removal of foley tubes, and intraoperative body temperature control as key measures to reduce SSI (Rosenberger et al., 2011). The use of AMP is crucial and its efficacy has been proved in many studies. First- or second-generation cephalosporins are recommended as first-line AMP by most guidelines due to satisfying cost-effectiveness. For patients with penicillin allergy (PA), vancomycin and clindamycin are usually recommended as alternatives (Bratzler et al., 2013; Martin et al., 2019). Moreover, vancomycin is also considered for patients with methicillin-resistant Staphylococcus aureus (MRSA) colonization (Goyal et al., 2013; Wyles et al., 2019).

The efficacy of alternative antibiotic prophylaxis is still debatable compared to that of first-line cephalosporins. Studies showed elevated risk for adverse outcomes and various complications (Jeffres et al., 2016; MacFadden et al., 2016; Huang et al., 2018; Kaminsky et al., 2022), including SSI for surgical candidates (Blumenthal et al., 2018). Most of the relevant orthopedic studies analyzed patients after joint procedures with controversial outcomes (Ponce et al., 2014; Tan et al., 2016; Wyles et al., 2019). The impact of having PA is not otherwise reported in the literature regarding spine, trauma, or bone tumor surgeries. Moreover, the pathogenic differences have not been elucidated based on the presence of PA. Therefore, we performed a comprehensive analysis on PA and SSI based on the orthopedic patients in our center.

## 2 Method

### 2.1 Study population and inclusion criteria

This is a single-center retrospective cohort study. After obtaining approval from the Institutional Review Board in our center (Protocol number: K0385), we performed analysis on patients admitted to the orthopedics department in Peking Union Medical Collage Hospital during 1st January 2015 and 31st December 2021. All data was extracted from the Big Data Query and Analysis System in our hospital. Patients with existing history of penicillin allergy were included as the PA group, defined as penicillin allergy in medical records, regardless of the phenotype (i.e. self-reported allergy, positive penicillin skin testing, hypersensitivity reactions, etc.), with patients without penicillin allergy as the control group. The exclusion criteria included: 1) preoperative history of local or systemic infection; 2) admission due to open trauma; 3) postoperative infection from previous surgery in other centers; 4) delayed surgical site infection; 5) lack of antimicrobial prophylaxis preoperatively.

### 2.2 Perioperative protocol

In patients without PA, cefuroxime was the first-line antibiotics administered preoperatively at a dose of 1.5g intravenously 60 minutes before skin incision. In patients with PA, vancomycin or clindamycin were the preferred alternative antibiotic choice (vancomycin: 30mg/kg i.v. 120 minutes before skin incision, and clindamycin: 0.6-0.9g i.v. 60 minutes before skin incision, respectively). All patients received standardized perioperative infection prevention measures besides AMP, including but not limited to: hand hygiene, skin antiseptics, laminar airflow, maintaining normothermia, glucose control, etc.

### 2.3 Study outcomes

The primary outcome of this study was SSI after orthopedic surgeries. We first identified potential SSI based on following features: infection-related diagnosis, positive pathogen cultures, any debridement surgery, and any non-scheduled secondary surgery within 180 postoperative days. After thorough examination of their medical records, the diagnosis of SSI was established based on the 2021 CDC-NHSN criteria. The secondary outcomes of this study were the deep SSI, superficial SSI, and the AMP regimens between the two groups. Lastly, we described the common pathogens found in our SSI patients.

The primary exposure variable in this study was the presence of penicillin allergy. Based on literature review, the following SSI-related confounding factors were collected: 1) patient factors: age, hypertension, diabetes, hyperlipidemia/hypercholesterolemia, smoking history, alcohol intake, and American society of Anesthesiologists (ASA) class; 2) surgical factors: surgical history of the same site, emergency surgery, long operation duration (>3 hours), internal fixation, and implantation of joint prosthesis.

Eight orthopedic surgery types were included in this study: spine, joint replacement, trauma, arthroscopy, bone tumor, foot and ankle, hand surgery, and other surgeries (core decompression, limb deformity correction, and amputation, etc.).

### 2.4 Statistical analysis

Normally distributed quantitative data were presented as mean ± standard error, quantitative data without normal distribution were presented as quartiles, and count data were shown as numbers and its proportions [N, (%)]. Absolute standardized difference was used to compare the baseline characteristics, and <0.1 was considered to be balanced. Levene test was used to test for equal variance. For primary outcome, we utilized logistic regression and propensity score (PS) analysis to evaluate the correlation between PA and orthopedic SSI. Four models were used in the regression analysis: Model 1) univariable analysis; Model 2) incorporating confounding patient factors into the regression model; Model 3) incorporating confounding surgical factors into the regression model; and Model 4) including all confounding factors into the regression. SPSS PS matching tool (Version 3.04) was used for PS analysis to match patients with and without PA in a 1:4 ratio, with a caliper width of 0.1. All confounding factors were included into the propensity score matching (PSM) as covariates. In the regression analysis model, the ASA score was dichotomized as ≤ Class II and >Class II, and operation duration as >3h and ≤3h.

We performed mediation analysis using bootstrap methods based on both Model 4 and PSM Model to evaluate if the correlation between PA and SSI was mediated by the choice of AMP regimens. AMP regimens were further divided into cephalosporin and non-cephalosporin ones to see the potential mediation effect of the latter in the statistical analysis.

Statistical analysis was completed on SPSS 23.0, and a two-sided p-value < 0.05 was considered as statistically significant.

## 3 Results

### 3.1 Patient characteristics

**Figure 1:**
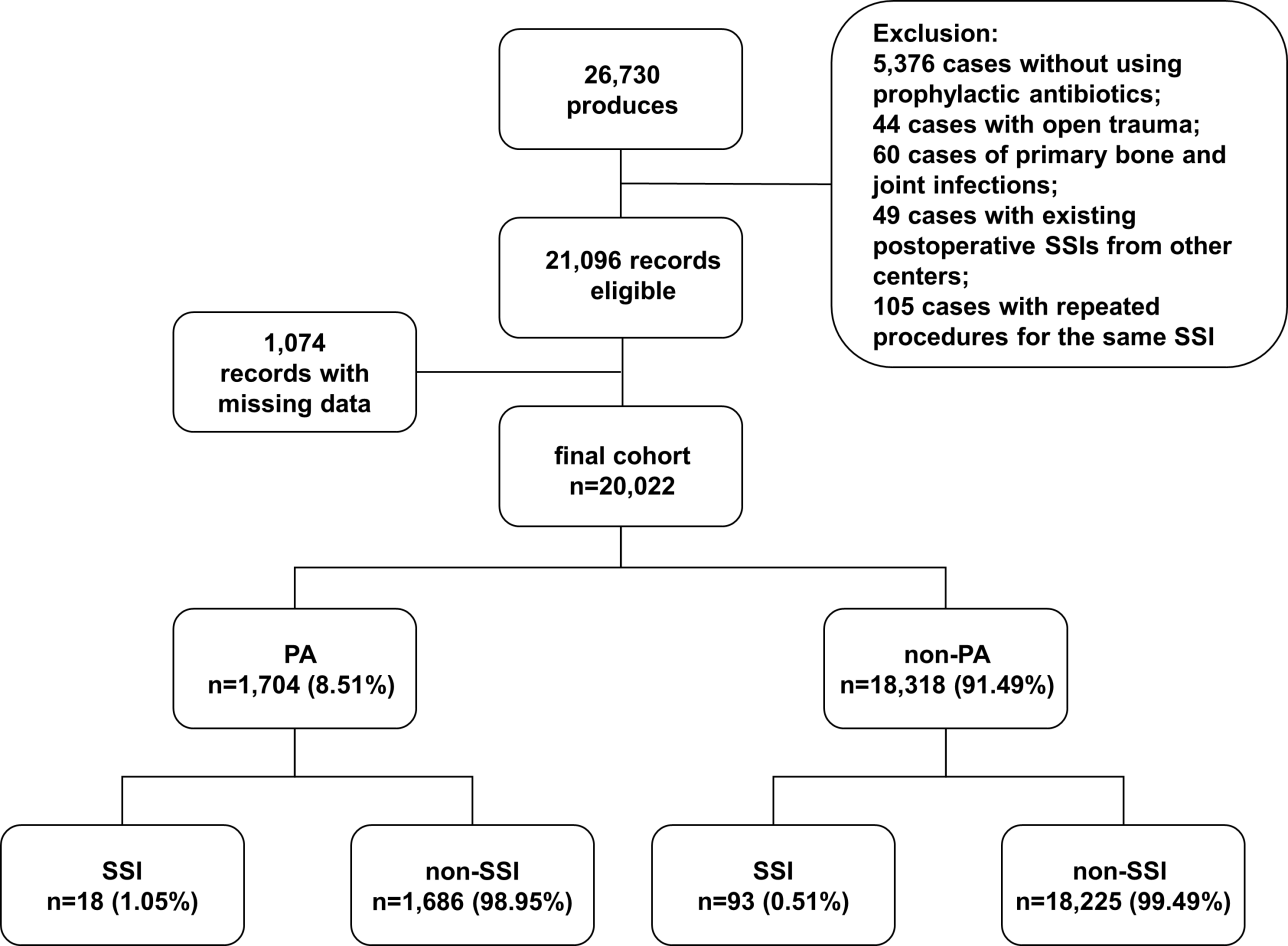
Flow diagram shows the study cohorts.

From January 2015 to December 2021, a total of 26730 surgical admission records were identified, and 20022 records met our inclusion criteria (**Figure 1**). Among them, 1704 records were with PA (8.51%, 1704/20022). Patients in PA group were generally older in age, predominantly female, and with less smoking history and alcohol intake. The patients in PA group also had higher prevalence of multiple comorbidities and higher ASA classes compared to non-PA group. Differences were also found in surgery types and the percentage of secondary surgery. After PS Matching, the baseline characteristics were balanced between the two groups (**Table 1**).

**Table 1:** Baseline Characteristics before and after propensity score matching.

Characteristic	Before Matching			After Matching		
	Penicillin Allergy Group (n=1704)	Non-Penicillin Allergy Group (n=18318)	Standardized Difference	Penicillin Allergy Group (n=1704)	Non-Penicillin Allergy Group (n=6786)	Standardized Difference
Age	56.3 (19.8)	50.6 (21.8)	0.274	56.3 (19.8)	56.3 (20.1)	0.000
Sex (Female)	1251 (73.4)	10855 (59.3)	0.302	1251 (73.4)	4945 (72.9)	0.011
Hypertension	689 (40.4)	5542(30.3)	0.212	689 (40.4)	2762 (40.7)	0.006
Diabetes	235 (13.8)	1973 (10.8)	0.091	235 (13.8)	942 (13.9)	0.003
Hyperlipidemia	108(6.3)	737 (4.0)	0.104	108(6.3)	431 (6.4)	0.004
Smoking	218(12.8)	2914 (15.9)	0.089	218(12.8)	804 (11.8)	0.032
Alcohol intake	21 (1.2)	488 (2.7)	0.108	21 (1.2)	138 (2.0)	0.064
ASA Class						
1	310 (18.2)	4781 (26.1)	0.191	310 (18.2)	1372 (20.2)	0.051
2	1148 (67.4)	11706 (63.9)	0.074	1148 (67.4)	4450 (65.6)	0.038
3	244 (14.3)	1794 (9.8)	0.138	244 (14.3)	948 (14.0)	0.009
4	2 (0.1)	37 (0.2)	0.026	2 (0.1)	15 (0.2)	0.082
Surgery type						
Spine	753 (44.2)	9397 (51.3)	0.065	753 (44.2)	2942 (43.3)	0.018
Joint replacement	526 (30.9)	4557 (24.9)	0.127	526 (30.9)	2046 (30.2)	0.011
Trauma	190 (11.2)	2016 (11.0)	0.006	190 (11.2)	896 (13.2)	0.058
Arthroscopy	73 (4.3)	662 (3.6)	0.036	73 (4.3)	275 (4.1)	0.010
Bone tumor	70 (4.1)	902 (4.9)	0.044	70 (4.1)	315 (4.6)	0.030
Foot and ankle	67 (3.9)	582 (3.2)	0.043	67 (3.9)	235 (3.5)	0.026
Hand	16 (0.9)	104 (0.6)	0.035	16 (0.9)	42 (0.6)	0.035
Others	9 (0.5)	98 (0.5)	0.000	9 (0.5)	34 (0.5)	0.000
Multiple surgical history of the same site	53 (3.1)	457 (2.5)	0.036	53 (3.1)	204 (3.0)	0.006
Emergency surgery	17 (1.0)	170 (0.9)	0.010	17 (1.0)	66 (1.0)	0.000
Internal fixation/joint prosthesis	1507 (88.4)	16005 (87.4)	0.031	1507 (88.4)	5965 (87.9)	0.015
Operation duration>3h	434 (25.5)	5639 (30.8)	0.118	434 (25.5)	1714 (25.3)	0.005

### 3.2 Primary outcome

#### 3.2.1 Correlation between PA and orthopedic SSI

A total of 111 (0.55%) SSI were identified after 20022 operations, 18 (1.05%) after 1704 operations in PA group, and 93 (0.51%) after 18,318 operations in the control group. Correlation between PA and orthopedic SSI was seen in both univariable analysis (OR 2.09; 95%CI, 1.26-3.47; p= .004) and multivariable logistic regression after including all confounding factors (OR 2.11; 95% CI, 1.26-3.50; p=0.004) (**Table 2**).

**Table 2:** Logistic regression and propensity score analysis of the association between penicillin allergy and orthopedics surgical site infection.

Logistic regression models (n=20022)	OR	95% Cl	p-value
Model 1 (Unadjusted)	2.09	1.26-3.47	0.004
Model 2 (patient-related confounders adjusted)	2.06	1.23-3.43	0.006
Model 3 (surgery-related confounders adjusted)	2.13	1.28-3.55	0.003
Model 4 (fully adjusted)	2.11	1.26-3.50	0.004
Propensity score analysis (n=8490)			
PS matching	1.84	1.05-3.23	0.034

OR, odds ratio; CI, confidence interval; PS, propensity score.

Model 1 was a univariable crude model;

Model 2 included age, hypertension, diabetes mellitus, hyperlipidemia, smoking history, drinking history, ASA classification;

Model 3 included multiple surgical history of the same site, emergency surgery, operation duration>3h, implantation-related surgery;

Model 4 includes all the above confounders.

After performing PSM, a total of 6786 surgical records were matched to the 1,704 records in PA group. Similarly, 39 (0.57%) SSI were found in the matched control group, compared to 18 (1.05%) in the PA group. The potential confounding factors were distributed evenly in the PA and control group after PSM. Regression analysis still showed correlation between PA and SSI (OR 1.84; 95% CI, 1.05-3.23; p= 0.034).

Mediation analysis based on Model 4 revealed no statistical significance in the direct effect of PA on SSI incidence (OR -0.002; 95% CI -0.008-0.003 p= 0.330), but elevated OR in the indirect effect of AMP choice (OR 0.008; 95% CI, 0.004-0.012; p= 0.002). Meantime, mediation analysis based on PSM Model also revealed no statistical significance in the direct effect of PA on SSI incidence (OR -0.004; 95% CI -0.011-0.003 p= 0.264), but elevated OR in the indirect effect of AMP choice (OR 0.009; 95% CI, 0.004-0.015; p< 0.001). Therefore, using non-cephalosporins as AMP has complete mediation effect on elevated SSI rate in PA patients.

### 3.3 Secondary outcomes

#### 3.3.1 Correlation between PA and SSI type

We then performed analysis on deep and superficial infections. Among the 20022 operations included, there were 52 (0.26%) superficial SSI, 6 (0.35%) in PA group, and 46 (0.25%) in non-PA group. Uni- and multi-variable analysis both demonstrated no relationship between PA and superficial SSI (OR 1.44; 95%Cl, 0.61-3.37; p= 0.404; adjusted OR 1.39; 95% CI, 0.59-3.29; p= 0.449).

Among the 59 (0.29%) deep SSI in our cohort, 12 (0.71%) were in PA group, and 47 (0.26%) in control group. Strong correlation between PA and deep SSI after orthopedic surgeries was seen both in univariable analysis (OR 2.70; 95%Cl, 1.43-5.09; p= 0.001) and multi-variable logistic regression with all confounders incorporated (OR 2.79; 95% CI, 1.47-5.30; p= 0.002).

#### 3.3.2 Preoperative AMP regimens

**Figure 2:**
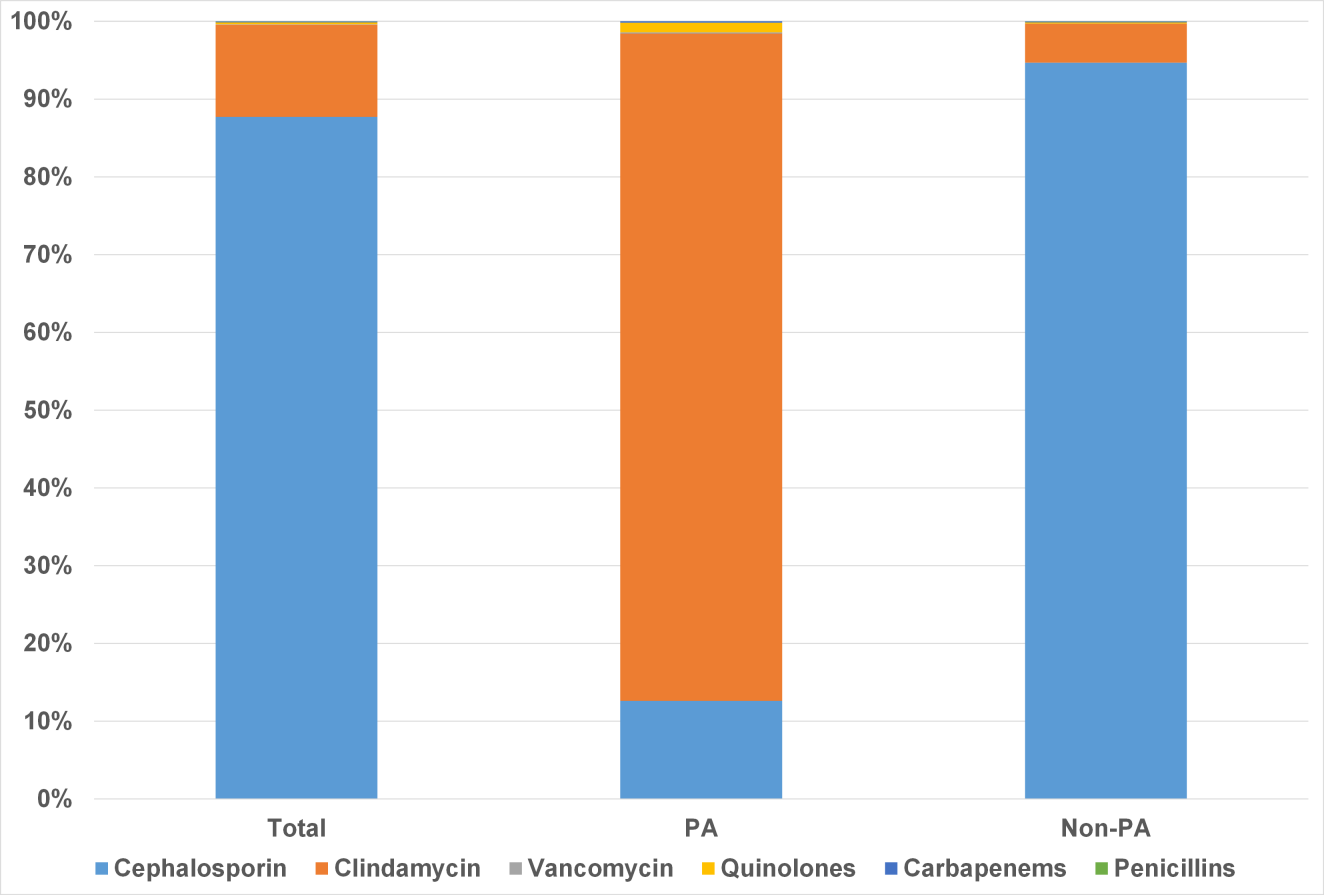
Preoperative prophylactic antibiotic regimens in patients with and without penicillin allergy.

Cefuroxime was the most commonly prescribed AMP in our cohort (n=17562, 87.71%), followed by clindamycin (n=2376, 11.87%). Patients with PA used significantly less cefuroxime (12.61%, 215/1704 vs. 94.69%, 17347/18318; p< 0.001), and more clindamycin (85.79%, 1462/1704 vs. 4.98%, 914/18318; p< 0.001) and quinolones (1.23%, 21/1704 vs. 0.15%, 27/18318; p< 0.001). Due to limited sample size, other alternative antibiotics, including vancomycin, were not further analyzed (**Figure 2**).

### 3.4 Pathogen characteristics

**Figure 3:**
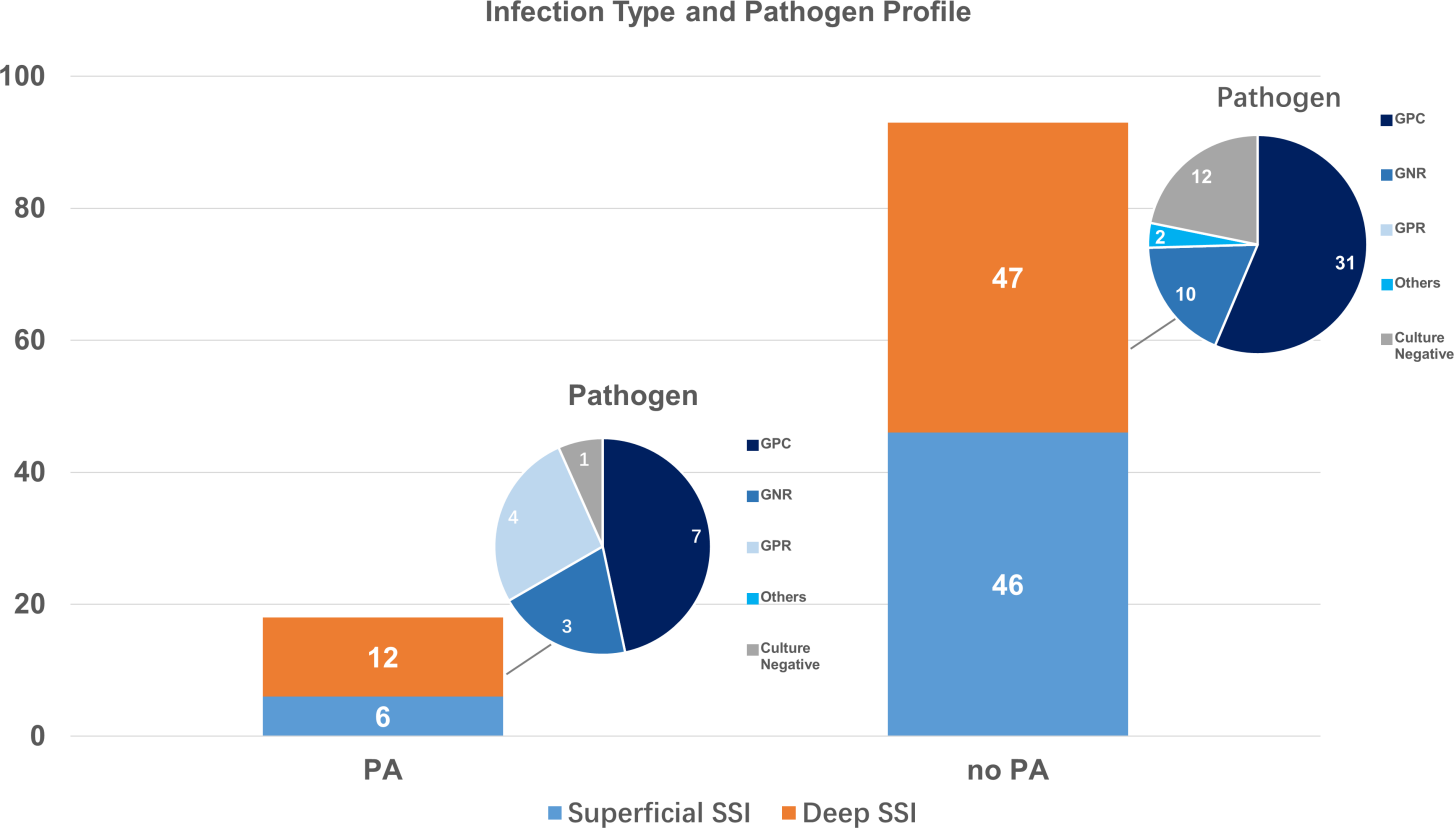
Pathogen profile of deep SSI in PA and Non-PA group.

Among the 111 SSI found in our study, 69 (62.16%) was caused by single-pathogen infection, 9 (8.11%) by multi-pathogen infection, and 33 (29.73%) had negative culture. Gram-positive cocci (GPC) were the leading pathogen for SSI, accounting for 69 (88.46%) cases among the 78 positive cultures. Gram-negative rods (GNR) and Gram-positive rods (GPR) accounted for 15 (19.23%) and 6 (7.69%) infections, respectively. Fungal and actinomycotic infection were found in 3 (3.85%) cases. Patients with and without PA had different pathogen profiles for deep SSI. Though GPC remained to be most commonly seen in deep SSI, patients with PA had higher infection rate from GPR and GNR than non-PA group (**Figure 3**).

## 4 Discussion

In this cohort study that included 20,022 patients undergoing various orthopedic surgeries, we found a higher proportion of postoperative SSI among patients with PA. Mediation analysis demonstrated possible effect of alternative antibiotic use on the SSI among PA patients. While previous studies mainly focused on arthroplasty, ours covers the spectrum of spine, joint, trauma, arthroscopy, bone tumor, foot and ankle, and hand surgeries. Our result suggests that the impact of having PA on SSI exists for various orthopedic procedures.

We also advanced our study by performing further analysis on infection type and pathogens. In our study, the influence of PA mainly caused increase in deep SSI instead of superficial SSI. This could be related to a higher proportion of internal fixation among orthopedic patients, where it inevitably affects peripheral blood supply, and the disadvantages of using alternative antibiotics would be magnified when dealing with microbes adhering to deep tissue or fixation device. Previous study reported higher SSI after alternative vancomycin prophylaxis due to its narrow spectrum mainly against Gram-positive microbes (Kheir et al., 2017). Our PA cohort mainly used clindamycin as alternative antibiotics, which has a broader spectrum compared to vancomycin. But the resistance to clindamycin from Staphylococcus aureus and Cutibacterium acnes was reported to elevate in recent years (Achermann et al., 2014; Wang et al., 2021). Other GNRs may have inherent resistance by limiting permeability of clindamycin through outer membranes, like the families of Pseudomonas, Enterobacillus, and Acinetobacter (Leclercq and Courvalin, 1991). These pathogens were all common culprits for deep SSI in our PA cohort. Additionally, as a bacteriostatic antibiotic, clindamycin requires much higher concentration to be bactericidal (Spížek and Řezanka, 2004). Despite its comparable bacteriostatic outcome with bactericidal antibiotics in therapeutic use (Wald-Dickler et al., 2018), the role of clindamycin is still to be studied when used prophylactically.

Some previous studies reported correlation between elevated SSI risk and PA with alternative antibiotic use. A study by Blumenthal et al. (Blumenthal et al., 2018) enrolled 8,385 patients from obstetrics and gynecology, general surgery, and orthopedics departments and showed an 50% elevated risk of SSI in PA patients compared to general population due to the use of alternative antibiotics. Reports in orthopedics mainly focused on patients after arthroplasty. Ponce et al. (Ponce et al., 2014) analyzed more than 18,000 patients and concluded that patients on vancomycin AMP developed significantly more SSI than patients on cefazolin. Regarding the argument of under-dosing and untimely use of vancomycin, Kheir et al. (Kheir et al., 2017) launched a study on AMP use and still revealed an elevated SSI rate in vancomycin group than in cefazolin group despite timely and full-dose administration. Other studies, however, found no correlation between PA and SSI. A study by Tan et al. (Tan et al., 2016) analyzed 10,391 patients receiving joint replacement, and showed no difference in infection between patients with and without PA, as well as reduced MRSA infection rate in patients receiving vancomycin AMP. Another study on joint replacement by Stone et al. (Stone A. et al., 2019) demonstrated similar SSI rate after arthroplasty in patients with and without PA. But this study had limited statistical power with a small sample size of 5,000 patients, given a low SSI rate (0.6% in PA group and 0.4% in general population). This conclusion has also been derived in other surgical fields, for example after colorectal surgeries as described by Khan et al. (Khan et al., 2021). Despite controversy on the correlation, surgeons should still prioritize cephalosporins as AMP due to its cost-effectiveness and relatively definite prophylactic efficacy.

The prevalence of PA is reported to be 5-25% in general population (Li et al., 2019; Stone C. et al., 2019). Recent study showed a lower cross-reactivity rate between penicillin and cephalosporins than previously perceived, with limited cases of true IgE-mediated reaction (Shenoy et al., 2019). A meta-analysis on 6,001 patients from 30 studies calculated a mere 0.7% of cross-allergy rate in patients who received cephalosporins despite their PA (Sousa-Pinto et al., 2021). Based on this result, multiple efforts have been developed to rule out unreliable PA labels, mainly through skin tests or provocation tests. But for surgical candidates, skin test and oral provocation could inevitably prolong hospital stay and incur medical costs, and our end goal here is simply safe administration of cephalosporins. Based on this clinical demand, Kuruvilla et al. (Kuruvilla et al., 2020) developed a streamlined history-based screening stratification without potential anaphylactic exposure to increase the cephalosporin use, and successfully resulted in 80% use among PA patients. Compared with additional or repeated skin testing, history-based screening might be more friendly for surgical departments to adopt to safeguard the use of β-lactam antibiotics.

There are several limitations to our study. First, around 5% of all admission records were excluded from this study due to missing data (mainly the ASA classification and operation time) of unknown mechanism. We did not compensate by filling in the missing data, which could result in impaired statistical power. Notably, however, the missing data was equally distributed in two groups and we had relatively large sample size. Hence the missing data should not cause deviation to the conclusion. Second, subgroup analysis was not performed based on different surgical types due to low SSI rate in our cohort. Studies focusing on different orthopedic surgical types and their SSI relation to PA history are warranted in the future. Third, as SSI is multi-factorial, the retrospective design of this study cannot incorporate all SSI-related confounding factors, for example the use of corticosteroids, immunosuppressants, or the presence of neoplasia, to exclude their influence on the result. Additionally, we were not able to obtain detailed labeling information due to limited database to perform subgroup analysis on the relationship between PA types and SSIs. Study of this kind could be further carried on in the future. The definition of our choice is based on clinical practice, where surgeons would predominantly base their antibiotic choice on the existence of previous PA labels, regardless of specific types. Therefore PA subtype variance should have little impact on the conclusion of our study. Moreover, the baseline characteristics were not fully balanced in this cohort study. To reduce the impact of this limitation, we have used 4 logistic regression models to include categorized patient factors and surgical factors separately into the analysis, and used PSM to further reduce the confounding effects. Therefore, prospective study of high quality is in need to further validate this conclusion.

## 5 Conclusion

In this study, we found an elevated SSI risk in patients with PA when compared to those without, especially deep SSI. This could potentially be related to the alternative antibiotics used in this population instead of cephalosporins. Based on the low incidence of cross-reactivity between penicillin and cephalosporins reported and the observed safety of using cephalosporins in patients with PA, we recommend detailed history-taking and skin tests when necessary, to remove inappropriate PA label and to reduce SSI risk.

## Data availability statement

The raw data supporting the conclusions of this article will be made available by the authors, without undue reservation.

## Ethics statement

The studies involving human participants were reviewed and approved by Peking Union Medical College Hospital Institutional Review Board. Written informed consent for participation was not required for this study in accordance with the national legislation and the institutional requirements.

## Author contributions

TN, JZ, and YW contributed to conception and design of the study. TN, YB, and ZL collected the related data and wrote the manuscript. TN, YZ, and ZL analyzed the data. All authors contributed to the article and approved the submitted version.
